# Pachydermodactily – the great imitator of arthritis: a case series

**DOI:** 10.3325/cmj.2022.66.164

**Published:** 2025-04

**Authors:** Iva Rukavina, Marijan Frković, Mario Sestan, Ivana Brnadic, Alenka Gagro, Suzana Ožanić Bulić, Marija Jelusic

**Affiliations:** 1 University Hospital Center Zagreb, University of Zagreb School of Medicine, Zagreb, Croatia; ^2^Faculty of Medicine, Josip Juraj Strossmayer University of Osijek, Osijek, Croatia ^3^Children’s Hospital Zagreb, Zagreb, Croatia

## Abstract

Pachydermodactily is a rare digital fibromatosis of unknown origin, mainly affecting adolescent boys. It presents as symmetrical, painless thickening of the soft tissues, usually around the proximal interphalangeal joints (PIP). Patients often experience delayed diagnoses, receive unnecessary treatments, or are misdiagnosed with chronic inflammatory arthritis. Although the exact cause remains unclear, pachydermodactyly may be associated with repetitive mechanical trauma, such as rubbing or interlacing the fingers, which can lead to secondary skin thickening. Treatment is often not required given its benign prognosis, although some patients ask for therapy due to the cosmetic impact of the condition. The aim of this study was to present the characteristics of seven patients diagnosed with pachydermodactily at pediatric rheumatology outpatient clinics in Zagreb. Additionally, we performed a comprehensive literature review of reported cases published from 1975 to 2024 using PubMed and Google Scholar. The primary symptom observed was swelling of the soft tissues around the PIP and metacarpophalangeal joints, with some patients presenting with hyperkeratotic plaques resembling knuckle pads. One patient experienced hand pain. Clinical examination and diagnostic workup were performed (laboratory tests specific for rheumatologic diseases, radiological tests such as joint ultrasound, x-ray or magnetic resonance imaging, or skin biopsy) to exclude other conditions with similar clinical features and etiologies, such as juvenile idiopathic arthritis. None of the patients met the criteria for juvenile idiopathic arthritis according to the classification criteria of the International League of Associations for Rheumatology. Increasing awareness of pachydermodactyly and achieving accurate diagnoses can reduce unnecessary diagnostic tests, treatments, and patient anxiety.

Pachydermodactyly is a rare condition characterized by digital fibromatosis of unknown origin, primarily affecting adolescent boys (1-4). It was first described by Bazex et al in 1973. The term is derived from the Greek words “*pachy*” (meaning thick), “*dermo*” (skin), and “dactyly” (finger). Interestingly, similar hand changes were noted as early as 1904 by Garrod (5,6). The condition manifests as symmetrical, painless swelling of the periarticular soft tissues, mostly affecting lateral and dorsal aspects of the proximal interphalangeal joints (PIP) (7-11).

The exact cause of PDD remains unknown, and the etiology is not fully understood. It is believed to result from excessive mechanical manipulation of the affected joints (12,13). Bardazzi et al proposed a classification of five types of PDD, as follows: 1. classical – several fingers affected; 2. localized – one finger is affected; 3. transgrediens – affecting areas of metacarpophalangeal (MCP) joints; 4. familial – patients with a family history of PDD; and 5. PPD associated with tuberous sclerosis (14). x-ray examinations do not reveal any bony or articular changes in these patients, while histological analysis of affected tissues shows hyperkeratosis and/or an increased number of collagen fibers (15). Although pachydermodactyly is considered rare, it is misdiagnosed in many cases. Misdiagnosis can lead to unnecessary, costly, or invasive medical procedures or inappropriate treatment for conditions the patient does not have. Therefore, it is crucial to identify its characteristics to differentiate it from other disorders that may cause swelling in the PIP joints, especially juvenile idiopathic arthritis (16,17). Currently, there are no established guidelines for the treatment of PDD, although treatment is often unnecessary due to its benign nature (18). In this study, we present clinical, laboratory, and radiological characteristics of seven male adolescents (aged 12 to 17 years) diagnosed with pachydermodactily and provide a literature review of the topic. A comprehensive literature search was conducted to identify relevant articles on pachydermodactily. The PubMed and Google Scholar databases were searched using the term “pachydermodactily.” The search yielded 126 articles. Non-English articles, abstract-only papers, systematic reviews, and other studies were excluded, leaving behind 86 case reports. After careful perusal of these case reports, 37 were excluded and 41 quality papers were included in the study (Figure 1).

**Figure 1:**
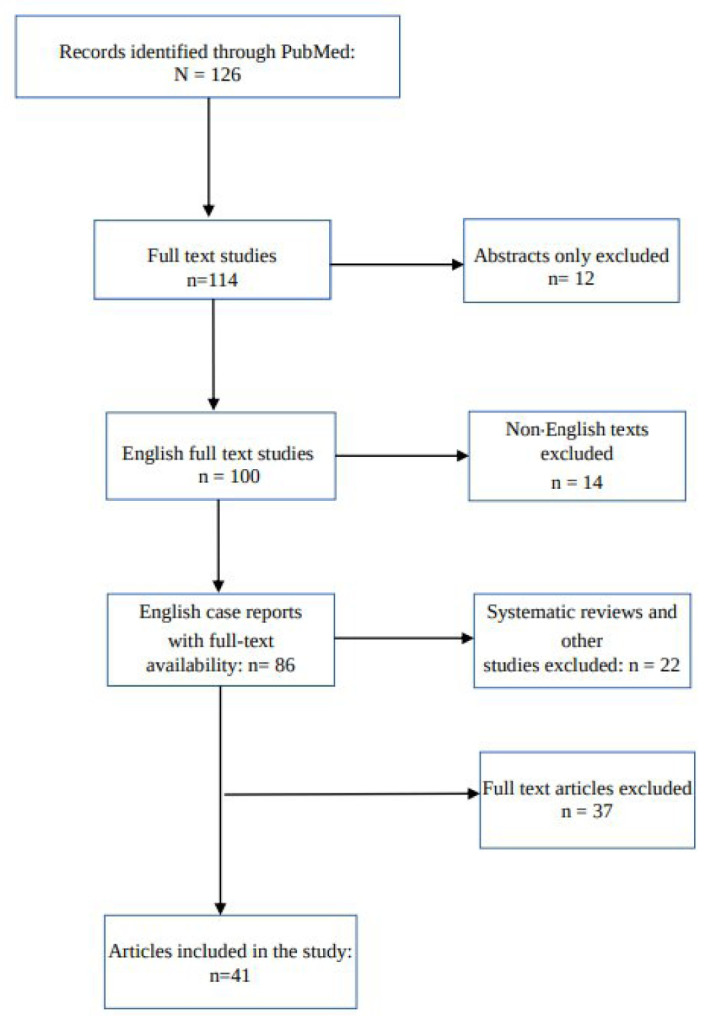
Flowchart of the systematic review on pachydermodactily in children.

We retrospectively reviewed the charts of patients with pachydermodactyly treated at the University Hospital Center Zagreb and Children’s Hospital Zagreb, Croatia, between 2015 and 2023. Patients were included if they had been followed for at least one year after diagnosis.

## Case 1

**Figure 2:**
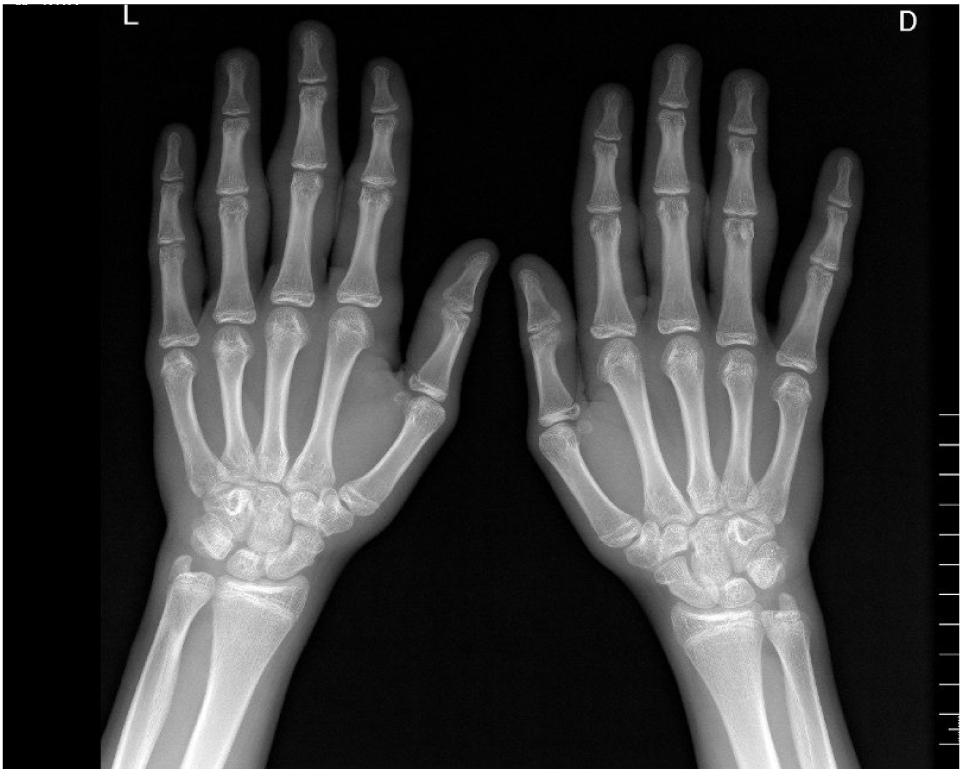
Periarticular thickening of the proximal interphalangeal joints bilaterally with discrete subcutaneous edema and no signs of synovitis.

A 16-year-old boy developed thickening of the second to fifth fingers at the level of the PIP joints bilaterally over the course of one year. The condition was painless, and the fingers were freely movable. There was no history of trauma or excessive manual activities, and the condition had remained unchanged since its onset. The patient reported no other illnesses or family history of similar issues. During the examination, Raynaud's syndrome was also observed. The patient's overall condition was good, and all relevant laboratory test results were within reference values, including markers for inflammation, serum levels of immunoglobulins, and the presence of antinuclear antibodies and rheumatoid factor. An ultrasonography of the hand joints showed normal findings, and x-rays of the hands did not reveal any involvement of the bone structures (Figure 2).

## Case 2

An 18-year-old boy had experienced progressive swelling in the PIP joints of the second to fifth fingers, as well as in the second and third MCP joints of both hands, over the previous six months. He also had hyperkeratotic plaques and nodules on the extensor surfaces of the affected joints. The patient felt pain in these joints, especially while writing. All laboratory tests, including complete blood count, biochemistry, coagulation, erythrocyte sedimentation rate, C-reactive protein, immunoglobulin and protein electrophoresis, rheumatoid factor, anti-cyclic citrullinated peptide (anti-CCP) antibodies, and antinuclear antibodies (ANA) were within the reference ranges. Hand x-rays and ultrasound examinations revealed soft tissue thickening around the PIP joints, but no signs of synovitis. Histopathological analysis showed hyperkeratosis, discrete perivascular inflammation in the papillary dermis, mucin deposits, and increased collagen bundles.

## Case 3

**Figure 3:**
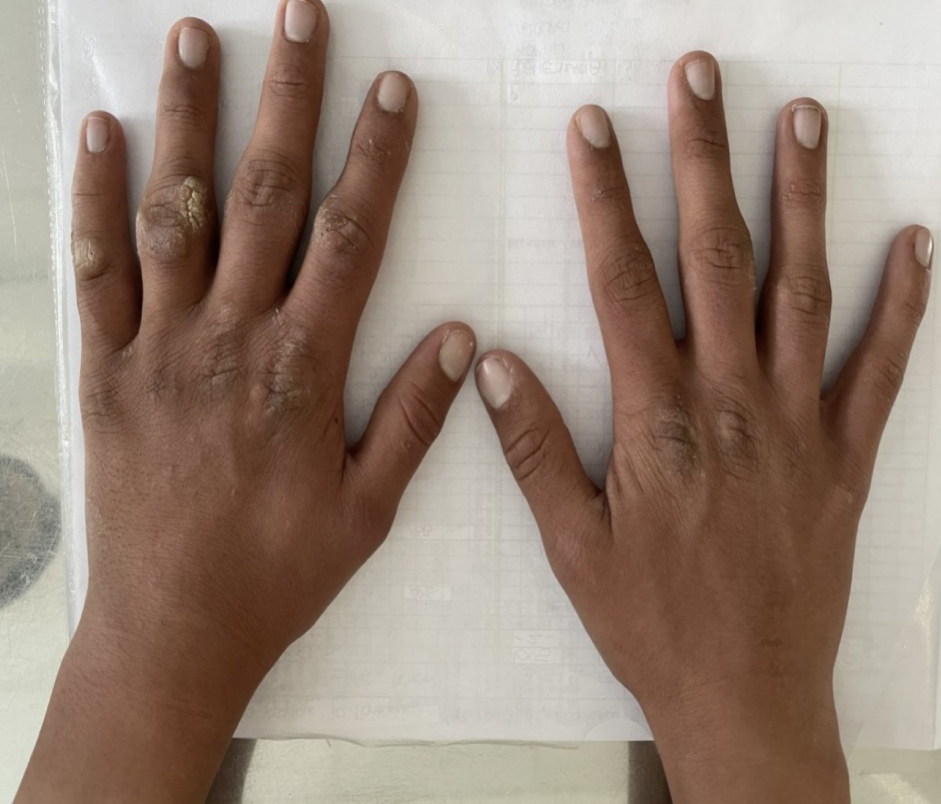
Deformity and swelling on the lateral surfaces of the proximal interphalangeal joints of the second, third, fourth, and fifth fingers with slightly keratotic plaques over the extensor side of the affected joints.

The case 3 was a 17-year-old boy with a congenital heart defect known as hypoplastic left heart syndrome, which was treated surgically with no long-standing complications, as well as Raynaud's syndrome. His initial presentation occurred two years before, when he was evaluated for symmetrical, progressive, and painless swelling in the PIP and MCP joints of the second to fifth fingers (Figure 3). He also exhibited hyperkeratotic plaques and nodules over the extensor areas of the affected joints. Importantly, he was not impaired in his daily activities, nor did he experience any limitations or tenderness during both active and passive range of motion assessments. Serological evaluations for inflammatory and connective tissue diseases returned negative results. A hand ultrasound revealed swollen soft tissues at the PIP and MCP joints bilaterally.

## Case 4

**Figure 4:**
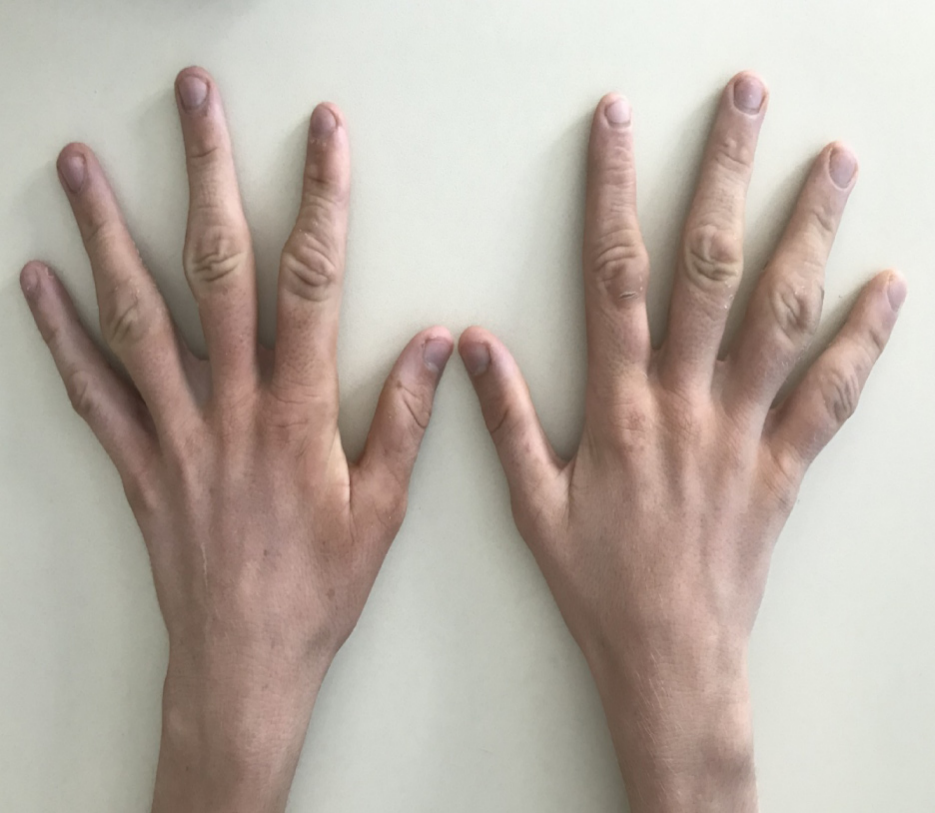
Bilateral thickening of the second to fifth proximal interphalangeal joint, with hyperkeratotic nodules and plaques, and some hyperkeratosis over the metacarpophalangeal joints.

**Figure 5:**
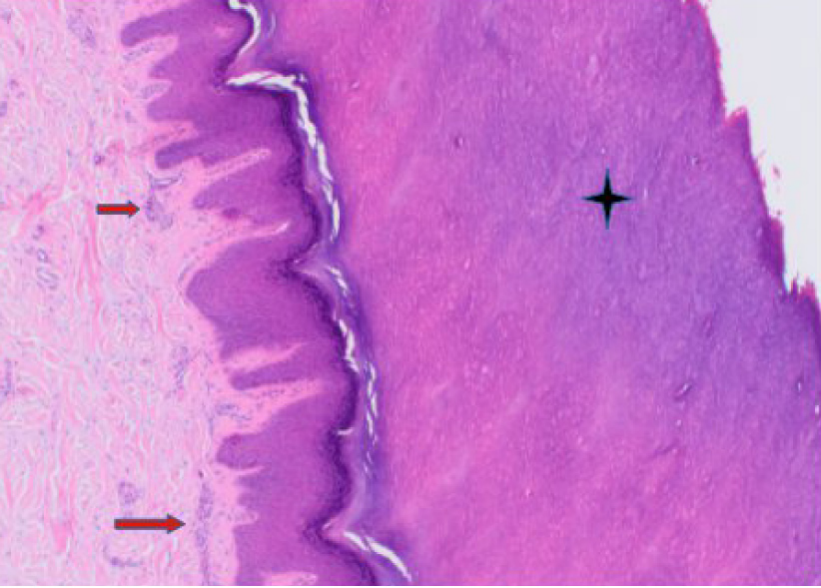
Histopathological analysis of the skin showing hyperkeratosis (four-pointed star) with discrete perivascular inflammation in the papillary dermis (red arrow).

**Figure 6:**
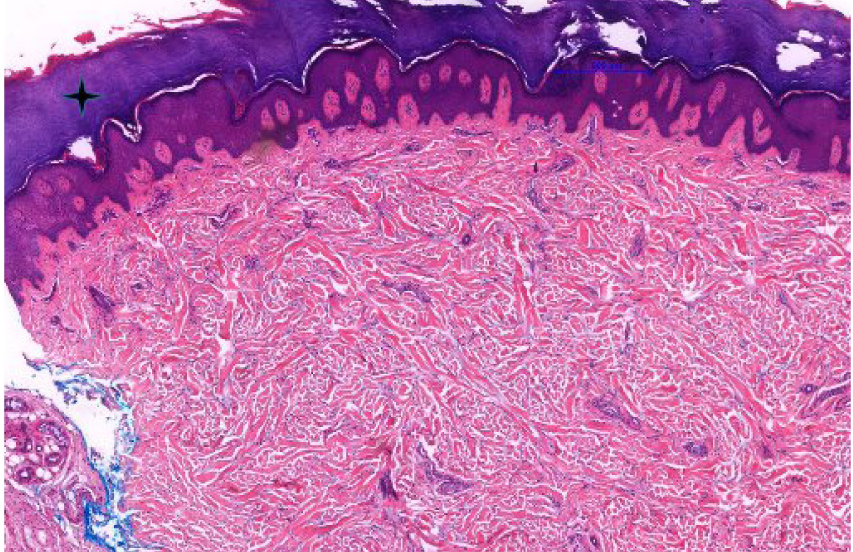
Histopathological analysis of the skin showing hyperkeratosis (four-pointed star) with proliferation of fibroblasts and increase in collagen bands in the dermis.

A 17-year-old boy presented with a two-year history of swelling in all PIP joints, as well as both thumb interphalangeal joints (Figure 4). Notably, no pain was associated with these joints. Additionally, the patient exhibited discrete nodular thickening of the skin at the extensor side of PIP joints, but there was no erythema, and joint movements remained within the normal range. He had been exposed to hard physical work and also displayed thoracic kyphosis. Importantly, there was no personal or family history of rheumatic diseases. Plain radiographs of the hands were normal, showing no erosions, periarticular osteopenia, periostitis, or cysts. An ultrasound of the hands revealed no synovial hypertrophy, vascularity, or bony erosions. Magnetic resonance imaging (MRI) of the hands showed no structural or inflammatory changes; the joint articular surfaces were preserved, and no erosive changes were detected. However, soft tissue thickening was observed. Histopathological analysis showed hyperkeratosis and perivascular inflammation in the papillary dermis (Figures 5 and 6).

## Case 5

A 16-year-old boy, with no family history of rheumatologic illnesses, presented with a two-year history of progressive, asymptomatic soft tissue enlargement in all the PIP joints of both hands. There was no tenderness or loss of range of motion. The skin overlying the joints was dry, occasionally exhibiting marked erythema and sometimes appearing eczematoid. He was diagnosed with contact allergic dermatitis and obsessive-compulsive disorder in terms of frequent finger rubbing. His complete blood count, RF, anti-CCP antibodies, ANA, erythrocyte sedimentation rate, and C-reactive protein levels were within the reference ranges. MRI of both hands showed soft tissue swelling without any bone involvement or loss of joint spaces. The histopathological examination revealed keratosis, acanthosis, and thickened collagen bundles in the dermis, along with a mild perivascular inflammatory infiltrate mainly consisting of mononuclear cells and mucin deposition between the collagen fibers.

## Case 6

A 12-year-old boy was referred to a pediatric rheumatologist for diagnostic evaluation due to suspected connective tissue disease. Three years earlier, thickening of the fingers was noted, but he had no other symptomatic complaints, and impaired hand function was not observed. During a physical examination, bilateral fusiform swelling was found on the lateral aspects of the PIP joints, accompanied by significant thickening of the skin and subcutaneous tissue. The skin over these lesions appeared rough and horny. Blood tests, including a complete blood count, percentage of white blood cells, and indicators of inflammation, were within the reference range. Autoantibody tests, including ANA antibodies and RF, returned negative results. As a result, a skin biopsy of the lesions was recommended for histopathological analysis. The biopsy revealed epidermal hyperplasia, hyperkeratosis of the skin, and sparse infiltrates of mononuclear cells around the blood vessels near the PIP joints.

## Case 7

A 17-year-old boy was referred to a rheumatologist for diagnostic evaluation due to suspected arthritis. During the previous year, he had experienced bilateral thickening of the second to fourth PIP joints in his left hand and the third to fourth PIP joints in his right hand. He did not complain of pain or morning stiffness, nor was he engaged in any activities that could have exacerbated this condition. He did not report any other complaints, and no additional abnormalities were found during the examination. Blood tests, including a complete blood count, white blood cell percentages, inflammatory markers (CRP and ESR), liver function tests, renal function parameters, and immunoglobulin levels, were all within the reference ranges. Additionally, both ANA and RF tests returned negative results. His mother suffered from arthritis.

## Diagnosis and treatment

Clinical observations – including interphalangeal skin thickening and, in some cases, hyperkeratosis – along with normal diagnostic test results pointed to the diagnosis of pachydermodactyly. Following this, we recommended increased skin care using emollient creams and topical corticosteroids for the affected areas of the skin. One patient used topical tacrolimus. All patients were advised to avoid physical labor. Additionally, we suggested intralesional triamcinolone injections; however, the patients declined this option due to the invasive nature of the treatment. After several years of follow-up, most patients remained stable and asymptomatic, except for one patient who initially experienced finger pain that has persisted but has not progressed to arthritis. The patient who was engaged in hard physical work continued working due to family circumstances. Patients who regularly used emollients noted slight improvements in their skin appearance, as well as those who applied topical corticosteroids or tacrolimus to inflamed skin areas. All patients provided informed consent for data publication.

## Discussion

Although pachydermodactyly is considered a very rare condition, it is likely more common than reported. To date, 134 cases have been documented in the literature, and we suspect that pachydermodactyly is significantly underdiagnosed. Since 2015, we have identified seven cases in two medical centers in Croatia, indicating that numerous undiagnosed cases may exist worldwide, or patients may be misdiagnosed with other conditions, particularly arthritis. This is why it is crucial to raise awareness about this condition (20). To the best of our knowledge, this represents the largest case series of pachydermodactyly ever published. It primarily affects male adolescents; however, a case of a 56-year-old woman has also been documented (1-4).

The etiology of pachydermodactyly is not yet understood, but it may be linked to mechanical trauma – such as repetitive rubbing of fingers seen in obsessive-compulsive disorder, atopic dermatitis, or during strenuous physical activity. Patients exhibit varying cutaneous responses to repetitive skin manipulation. In contrast to lichenification observed in atopic dermatitis, the skin overlying the affected areas in pachydermodactyly generally remains intact; however, erythema, desquamation, or hyperkeratosis may be present. We identified all of these triggers in three of our patients, while two of them had Raynaud syndrome. Three patients had no apparent cause (1,13). The average time between the onset of symptoms and diagnosis was 18 months, which is a significant period of time (Table 1). According to the literature, in some patients, time to diagnosis was as long as three years (3).

**Table 1:** Demographic and clinical characteristics of our patients

Patient	Age	Sex	Presentation	Time from symptom onset to diagnosis	Background
1	16	Male	Lateral swelling and thickening of the 2-5 PIP joints bilaterally	1 year	Raynaud syndrome
2	18	Male	Lateral swelling and thickening of the 2-5 PIP, 2-3 MCP and thumb interphalangeal joints bilaterally with pain while writing	6 months	None
3	17	Male	Lateral swelling and thickening, and hyperkeratotic nodulations at the extensor side of the 2-5 PIP and MCP joints bilaterally	2 years	Congenital heart defect treated surgically, Raynaud syndrome
4	17	Male	Lateral swelling and thickening, and hyperkeratotic nodulations at the extensor side of the 2-5 PIP joints together with nodular skin thickening and IP thumb joint bilaterally	2 years	Hard physical work
5	16	Male	Lateral swelling and thickening, and hyperkeratotic nodulations at the extensor side of the 2-5 PIP joints bilaterally together with eczematoid skin	0.5 years	Contact allergic dermatitis, obsessive compulsive disorder
6	12	Male	Lateral swelling and thickening, and hyperkeratotic nodulations at the extensor side of the 2-5 PIP joints bilaterally	3 years	None
7	16	Male	Lateral swelling and thickening of the 2-4 PIP of the left hand and 3-4 PIP joints of the right hand	1.5 years	None

*Abbreviations: IP – interphalangeal joints, PIP – proximal interphalangeal joints, MCP – metacarpophalangeal joints.

Generally, most PDD patients do not feel pain and have normal hand function, even though some sporadic cases felt pain, as was observed in one of our cases. These patients require close monitoring in order to recognize the developing of arthritis or any other disease in time, as both could coexist (1,10,11,20,21). Unilateral presentation in the affected joints is very rarely reported (12).

None of our patients had family members with similar symptoms, yet pachydermodactyly has been noted in some families, which suggests potential genetic factors (1). Furthermore, given that it predominantly affects young men, hormonal influences may contribute to the disease's development. Fortunately, boys typically tend to care less about the appearance of their hands compared with girls, which may help them cope better with the cosmetic aspects of the condition (19). Radiographic imaging revealed soft tissue swelling without loss of joint space, periarticular osteoporosis, periostosis, erosion, or osteophytes. MRI findings showed T1 hypointense, mildly T2 hyperintense, and minimally enhancing cutaneous thickening, with no evidence of joint effusion, synovitis, or tendinitis (22-24). Skin biopsies indicated thickened collagen bundles in the dermis, along with acanthosis, hyperkeratosis, and sparse mucin deposition, as was seen in our patients who underwent skin biopsy (25). There is currently no consensus on whether to perform arthritis screening or other diagnostic tests initially, but the clinical picture together with medical history should direct the physician to the right diagnosis. It seems prudent to conduct as few diagnostic tests as possible, not only for cost-saving reasons but also to avoid exposing patients to unnecessary procedures that may pose health risks (like x-rays) or pain from invasive procedures, such as skin biopsies. We followed this approach in several of our patients, especially in those where we lacked experience in diagnosing pachydermodactyly (26).

Pachydermodactyly should be considered in patients exhibiting progressive soft tissue swelling on the lateral aspect of the PIP joints, particularly when there is an absence of pain, tenderness, or functional impairment. It may also affect distal interphalangeal (DIP) and MCP joints (7), and there is even a documented case in which thumbs, DIP, and metatarsophalangeal joints were involved. One of our patients had interphalangeal thumb joints affected (7-11). Diagnosing pachydermodactyly often requires excluding other potential conditions, particularly in patients who do not display symptoms of other medical issues with similar clinical features. Pachydermodactyly can easily be misdiagnosed as inflammatory arthritis since many physicians may be unfamiliar with this disease (27), likely due to the limited number of documented cases in the literature (28). The differential diagnosis for pachydermodactyly is extensive, with juvenile idiopathic arthritis being the primary concern. A painless course with preserved joint function and normal blood tests typically rules out inflammatory arthropathy. Pachydermodactyly is often confused with knuckle pads, which occur predominantly on the dorsal aspects of the MCP joints, or pseudo-knuckle pads (also known as chewing pads in children). Importantly, these conditions may coexist. Furthermore, knuckle pads generally appear from the fifth decade of life onward, which makes them less likely in younger patients (25). Other conditions that may be considered in the differential diagnosis include the Thiemann disease, juvenile fibromatosis, thyroid acropachy, pachydermoperiostosis (the Touraine-Solente-Golé syndrome), tophi, acromegaly, subacute cutaneous lupus erythematosus, limited scleroderma or diffuse systemic sclerosis, progressive nodular fibrosis of the skin, foreign-body granuloma, fibrosing inflammatory conditions, paraneoplastic acropachydermodactyly, and hypertrophic pulmonary osteoarthropathy. Careful medical history taking and a thorough patient examination are essential to confirm the correct diagnosis (17).

Recommendations for treating patients with this condition need to be developed, as there are several therapeutic options available that different physicians employ based on their experiences and preferences. All of them are symptomatic. Many patients experience hyperkeratosis, so increased skin care is advised as a primary measure. This includes using lotions and creams containing urea, syndet soap for washing hands, and various emollients. If there are any inflamed skin areas, topical corticosteroids and calcineurin inhibitors such as tacrolimus and pimecrolimus could be helpful. There have been attempts to treat thickened skin with intralesional corticosteroid injections, with triamcinolone hexacetonide showing some potential for improvement, although results remain inconclusive (29-33). Surgical resection is another option (21,31). According to the available literature, there have been no recurrences of lesions following a short follow-up period after surgical resection (34). However, our patients were not interested in any invasive treatment. Given its benign nature, such a decision seemed quite reasonable. Patients need to be reassured that lesions tend to be stable for a long period of time or even get smaller. Even though the clinical picture may look impressive, patients need to understand that the condition poses no threat to their overall health (18,34-37). Increased awareness of this condition is vital to prevent misdiagnosis and unnecessary immunosuppressive treatments (18,38,39).
